# Supplementary material for: Copolymerization of Styrene and Pentadecylphenylmethacrylate (PDPMA): Synthesis, Characterization, Thermomechanical and Adhesion Properties
Source: Polymers (Basel). 2020 Jan 4;12(1):97. doi: 10.3390/polym12010097 (PMC7023656; doi:10.3390/polym12010097)
Supplement: Supplementary file 1 [file polymers-12-00097-s001.pdf]

polymers
MDPI

Supplementary Materials

Copolymerization of Styrene and Pentadecylphenylmethacrylate (PDPMA): Synthesis, Characterization, Thermomechanical and Adhesion Properties

Tomy Muringayil Joseph[1*], Sumi Muralidharan Nair[2], Suresh Kattimuttathu Ittara[2], Jozef T. Haponiuk[1*] and Sabu Thomas[3]

1 Chemical Faculty, Polymers Technology Department, Gdansk University of Technology, Gdansk, 80-233, Poland.
2 Polymers & Functional Materials Division, CSIR-Indian Institute of Chemical Technology, Hyderabad – 500 007, India
3 School of Chemical Sciences, Mahatma Gandhi University, Kottayam, India
* TomyMuringayil Joseph: tomymuringayiljoseph@gmail.com; Tel.: +48505440911; Józef. T. Haponiuk jozef.haponiuk@pg.edu.pl ;Tel.: +48583472134

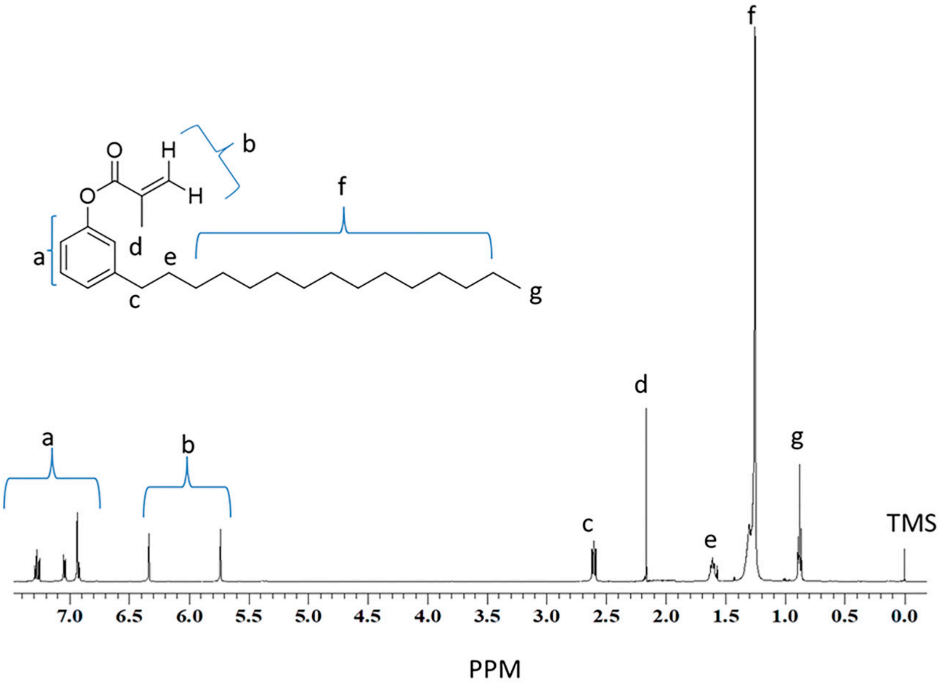

Figure S1. ^{1}H NMR spectrum of PDPMA monomer.

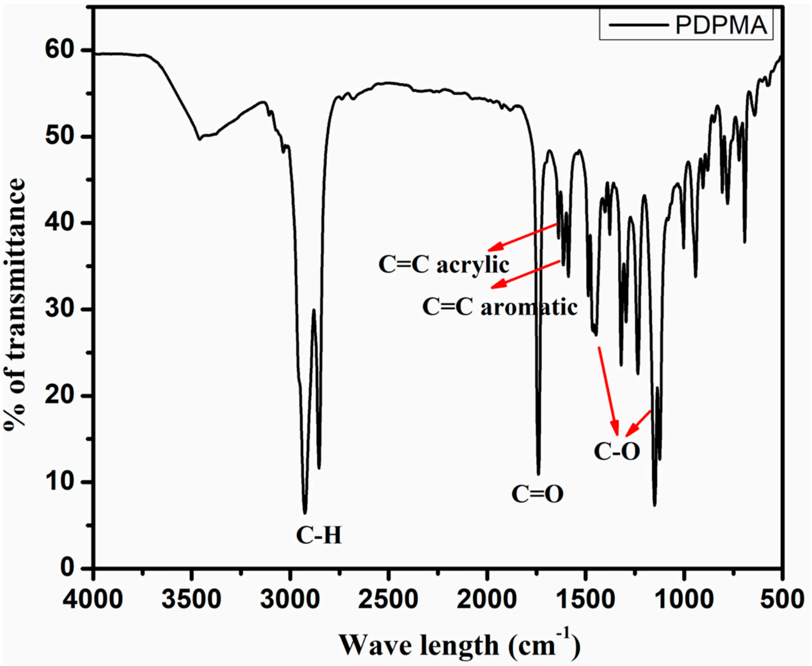

Figure S2. FT-IR spectrum of PDPMA monomer.

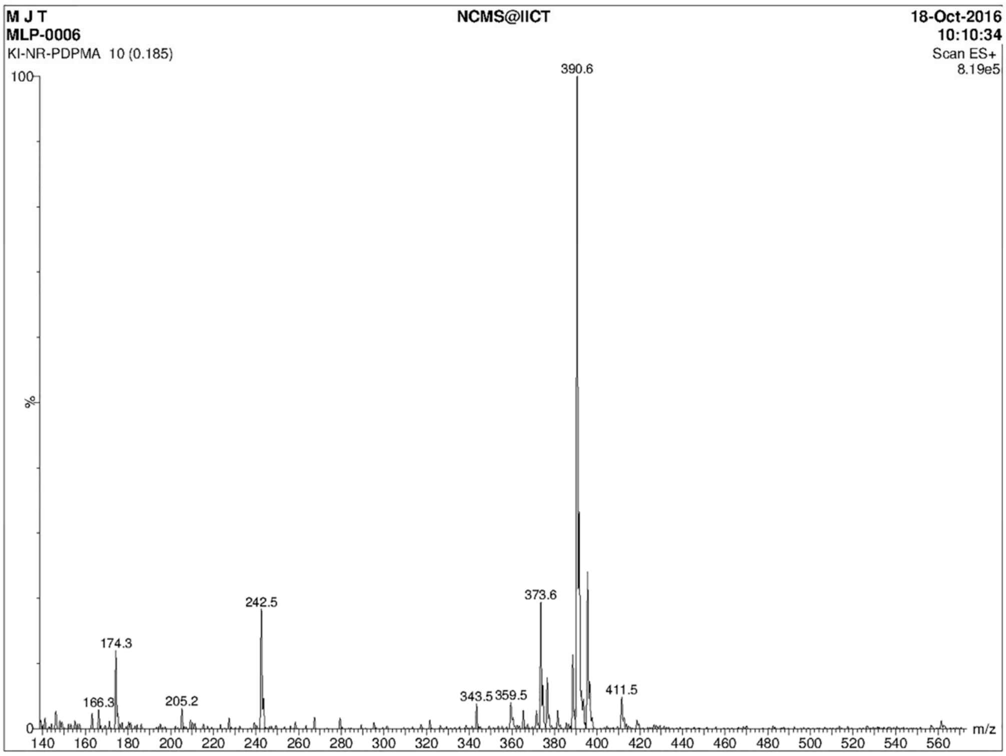

Figure S3. Mass Spectrum of PDPMA monomer.
